# Preclinical specificity & activity of a fully human 41BB-expressing anti-CD19 CART- therapy for treatment-resistant autoimmune disease

**DOI:** 10.1016/j.omtm.2024.101267

**Published:** 2024-05-20

**Authors:** Binghao J. Peng, Andrea Alvarado, Hangameh Cassim, Soprina Guarneri, Steven Wong, Jonathan Willis, Julia SantaMaria, Ashley Martynchuk, Victoria Stratton, Darshil Patel, Chien-Chung Chen, Yan Li, Gwendolyn K. Binder, Rebecca Dryer-Minnerly, Jinmin Lee, Samik Basu

**Affiliations:** 1 Department of Cellular and Molecular Immunology, Cabaletta Bio, Philadelphia, PA 19130, USA; 2 Department of Protein and Molecular Biology, Cabaletta Bio, Philadelphia, PA 19130, USA; 3 Department of Analytical Development, Cabaletta Bio, Philadelphia, PA 19130, USA; 4 Department of Manufacturing, Science, and Technologies, Cabaletta Bio, Philadelphia, PA 19130, USA; 5 Cabaletta Bio, Philadelphia, PA 19130, USA

**Keywords:** chimeric antigen receptor T cell, CAR T cell, autoimmune disease, preclinical study

## Abstract

Over 4% of the global population is estimated to live with autoimmune disease, necessitating immunosuppressive treatment that is often chronic, not curative, and carries associated risks. B cells have emerged as key players in disease pathogenesis, as evidenced by partial responsiveness to B cell depletion by antibody-based therapies. However, these treatments often have transient effects due to incomplete depletion of tissue-resident B cells. Chimeric antigen receptor (CAR) T cells targeting B cells have demonstrated efficacy in refractory systemic lupus erythematosus. To this end, we developed an anti-CD19 CAR T cell product candidate, CABA-201, containing a clinically evaluated fully human CD19 binder (IC78) with a 4-1BB costimulatory domain and CD3 zeta stimulation domain for treatment refractory autoimmune disease. Here, we demonstrate specific cytotoxic activity of CABA-201 against CD19^+^ Nalm6 cells with no off-target effects on primary human cells. Novel examination of CABA-201 generated from primary T cells from multiple patients with autoimmune disease displayed robust CAR surface expression and effective elimination of the intended target autologous CD19^+^ B cells *in vitro*. Together, these findings support the tolerability and activity of CABA-201 for clinical development in patients with autoimmune disease.

## Graphical abstract

**Figure:**
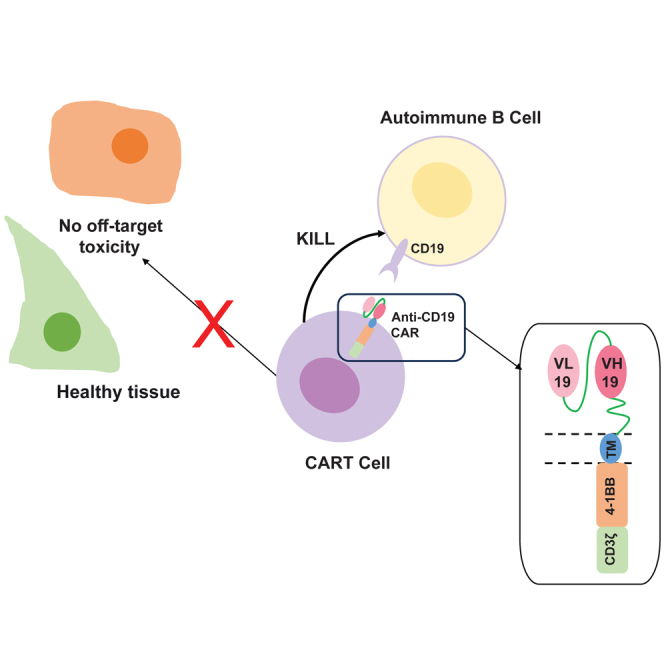

## Teaser

Basu and colleagues show CABA-201, a B cell-targeting CAR T cell product with a fully human CD19 binder and 4-1BB costimulatory domain, can precisely eliminate autoimmune patient B cells without off-target deleterious effects, demonstrating its ability as a robust therapeutic for B cell-driven autoimmune disorders.

## Introduction

Autoimmune disease is a major medical concern for a significant part of the world population. It comprises a heterogeneous group of medical conditions characterized by a shared etiology of the adaptive immune system targeting normal tissue antigens in concert with a breakdown in immune tolerance mechanisms.^1^ Such reactivity may be confined to organ-specific damage such as in autoimmune hepatitis or Hashimoto’s autoimmune thyroiditis and can also result in systemic manifestations such as are experienced with systemic lupus erythematosus (SLE) or rheumatoid arthritis (RA). The ability to identify and diagnose autoimmune diseases has evolved significantly over the last quarter century, leading to an increase in the number identified from 30 to over 100 described today.^2^^,^^3^ US-based disease prevalence ranges widely from extremely rare (e.g., fewer than 200 cases) to common (e.g., >2.5 million cases).^1^ An estimated 4.5% (6.4% females and 2.7% males) of the worldwide population has autoimmune disease, and approximately 14 million people in the United States are living with autoimmune disease.^1^^,^^4^ Autoimmune disease represents a major global unmet medical need for which new therapies are urgently needed.

Treatment for autoimmune disease has fallen primarily to systemic therapies including metabolic inhibitors (methotrexate and mycophenolate mofetil), immune suppressants (cyclosporine and corticosteroids), and cytotoxic therapies such as cyclophosphamide for severe manifestations. These therapies are a cornerstone for disease management; however, broad immune suppression comes with associated risks of infection and increased malignancy risk, as well as other non-immune-related side effects. Recently, the utilization of the B cell-depleting therapies, such as rituximab, have offered meaningful improvement in acute disease outcomes and have reduced comorbidities in certain autoimmune diseases.^5^ While clinical data on rituximab efficacy continue to emerge, evidence indicates that the therapy is non-curative for many patients despite prolonged administration. The failure is due, in part, to incomplete B cell depletion in lymphoid compartments.^6^ There is increasing evidence that B cells play a pathogenic role in diseases classically recognized as being T cell mediated such as type 1 diabetes, multiple sclerosis (MS), and RA based upon their partial responsiveness to anti-CD20 therapy especially in early phases of disease.^7^^,^^8^^,^^9^ Although the depletion of CD20^+^ CD8 T cells cannot be wholly excluded from an evaluation of anti-CD20 therapy efficacy, the contribution of these unique T cells in autoimmune pathogenesis is unclear.^10^^,^^11^ While primarily the source of the autoantibodies, autoreactive B cells also contribute to T cell-mediated autoimmunity through increased production of pro-inflammatory cytokines and upregulated major histocompatibility complex class II antigen presentation.^12^ Thus, it is possible that deep depletion of B cells in patients may lead to cessation of autoimmune disease by removing a central driver of autoimmune-induced inflammation (autoreactive B cells) and allowing the immune system to return to a tolerant state, resulting in deep and durable remissions off therapy.

The application of B cell-directed CD19 chimeric antigen receptor (CAR) T cell therapy offers to overcome the durability limitations of antibody-based B cell depletion and potentially deliver lasting responses. Anti-CD19 CAR T cells routinely traffic throughout the body, including into secondary lymphoid tissue where target B cells reside, and have demonstrated the systemic eradication of B cells in these tissues.^13^

CAR T cells are T cells that have been genetically engineered with a synthetic receptor that redirects the T cell to a cell surface antigen on a target cell. The synthetic CAR receptor is typically composed of an extracellular ligand-binding domain (typically a monoclonal single-chain variable [scFv] antibody fragment), optionally fused to a linker, a transmembrane domain, and intracellular T cell receptor signaling and costimulatory domains; variations on design can modulate T cell activation and function.^14^ CD19-directed CAR T cells have led to lasting remissions of B cell leukemias and lymphomas.^15^^,^^16^^,^^17^^,^^18^ Anti-CD19 CAR T cell therapies were the first CAR T cells to receive FDA approval with four now commercially available for patients: tisagenlecleucel (Kymriah), axicabtagene ciloleucel (Yescar Ta), brexucabtagene autoleucel (Tecar Tus), and lisocabtagene maraleucel (Breyanzi).^19^ These approved cell products all contain the same scFv binding domain, FMC63, which is derived from a murine CD19-specific monoclonal antibody. They also include the CD3ζ T cell activation domain and either CD28 or 4-1BB costimulatory signaling domains.

The CABA-201 CAR construct (CABA19-IC78) is composed of a CD8α signal peptide, a fully human anti-CD19 scFv containing a GS linker connecting the variable light and heavy chains,^20^ a portion of human CD8α hinge and transmembrane domain, the T cell activation domain CD3ζ, and costimulatory domain CD137 (4-1BB) (Figure S1). The fully human anti-CD19 binder was selected to minimize the potential immunogenicity of the CAR T cells, which could impact efficacy if repeat infusions are needed.^21^ CAR T cells containing the fully human anti-CD19 IC78 scFv have demonstrated similar properties and *in vivo* anti-tumor activity compared to the standard anti-CD19 FMC63-containing CAR T cell that has been extensively clinically tested and FDA approved.^22^ Here, we demonstrate the specific efficacy and safety of the CABA-201, the comparable activity of CABA-201 to FMC63 CAR T cells both *in vitro* and *in vivo*, which thus links the expected performance of CABA-201 in patients with autoimmune diseases to that observed in the pilot clinical studies,^23^^,^^24^^,^^25^^,^^26^ and absence of off-target effects *in vitro*. Most notably, we evaluated the ability of CABA-201 generated from the T cells of patients with various autoimmune diseases, including SLE, mucocutaneous pemphigus vulgaris (mcPV), MS, and RA, to target donor-matched autologous B cells. Collectively, the results provided here supported multiple CABA-201 investigational new drug applications and served as the nonclinical basis for the risk/benefit assessment required for the first-in-human clinical trials for CABA-201 CAR T cell therapy in patients with autoimmune disease.

## Results

### Cytotoxicity of CABA-201 toward CD19^+^ target cells

**Figure 1:**
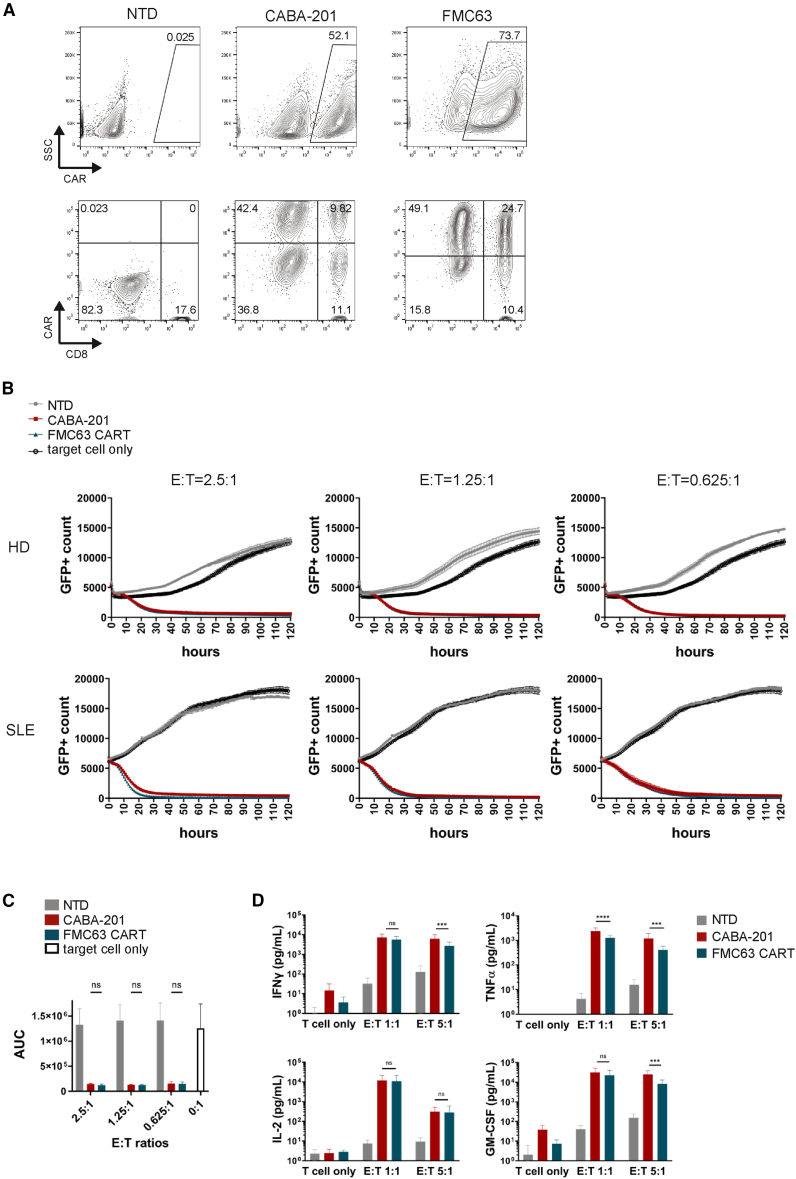
CABA-201 shows potent and specific cytotoxicity toward CD19^+^ cells (A) Characterization of CABA-201 and donor-matched FMC63 CAR T cells generated using a clinical-scale manufacturing process used in subsequent assays. Representative images from one donor are shown. (B) Effector cells (CABA-201, FMC63 CAR T, or NTD) were co-incubated with target Nalm6 cells for 120 h at indicated E:T ratios to measure specific cytotoxicity against CD19^+^ target Nalm6 cells expressing GFP. Cell death is indicated by the decrease in GFP^+^ count, shown as mean ± SD in triplicates. Representative graphs are shown for a healthy donor (HD) and SLE donor. Similar results were obtained from the other donors. (C) Area under the curve (AUC) is shown as mean ± SD from graphs in (B) and from the other donors (*n* = 4). (D) IFNγ, TNFα, IL-2, and GM-CSF production by CAR T cells in the supernatants collected after 24 h of CABA-201 from four donors co-cultured with CD19^+^ Nalm6 cells is shown. Donor-matched NTD and FMC63 CAR T cells were used. Each bar represents mean ± SD of four different donors. Statistical differences between CABA-201 and FMC63 CAR T cells were determined by two-way ANOVA with Tukey’s multiple comparisons test. *∗∗∗∗p* ≤ 0.0001, ∗∗∗*p* ≤ 0.001, ∗∗*p* ≤ 0.01, ∗*p* ≤ 0.05, ns = not significant. Experiments were repeated two times independently.

CABA-201 is a CD19-targeting, fully human, CAR T cell constructed using the same technology as an approved CAR T cell therapy, tisagenlecleucel. CABA-201 employs a fully human CD19 binder that has demonstrated similar activity to FMC63, the binder expressed on the CAR from the SLE study,^23^ as well as tisagenlecleucel. The selection of IC78, a fully human anti-CD19 binder, was made with the intent of reducing the chances of immunogenicity toward CABA-201 CAR T cells, which could impact their efficacy in the setting of reinfusion.^21^ In addition, the 4-1BB costimulatory domain and the CD3ζ activating domain are identical in the two approved CAR T cell products. *In vitro* and *in vivo* studies have demonstrated that 4-1BB and TCR/CD3ζ combination of costimulatory domains confers superior safety, efficacy and improved persistence to T cells expressing a CAR.^27^^,^^28^^,^^29^ We generated CABA-201 from four donor apheresis materials (three from healthy donors and one from an SLE donor) using a clinical scale process, and CABA-201 cells show comparable CAR expression and CD4/CD8 phenotype as donor-matched FMC63 CAR T cells (Figure 1A). CD19-specific CABA-201 cytotoxicity was assessed using the IncuCyte live-cell imaging system. CABA-201 cells demonstrated specific cytotoxicity toward Nalm6 cells endogenously expressing CD19 (Figures 1B and 1C), and their cytotoxic activity was comparable to donor-matched FMC63 CAR T cells, including cells manufactured from SLE donor material. The data indicated that both FMC63 CAR T and CABA-201 displayed more than 70% of cytotoxic activity over the non-transduced (NTD) and Nalm6 controls across all tested effector to target cell (E:T) ratios (Figure 1C). Growth kinetics of target only and NTD controls varied over the measured period, likely reflecting variation in basal level of cytokine production from NTD T cells aiding in target cell growth and allogeneic effects from each donor in addition to other potential donor specific differences. CABA-201 demonstrated comparable cytotoxic activity against Nalm6 cells to FMC63 CAR T cells across the donors and the E:T ratios tested. To assess cytokine secretion of CABA-201 in response to co-culture with CD19^+^ Nalm6 cells, 24-h co-culture studies were performed with CABA-201 or donor-matched FMC63 CAR T cells. CABA-201 and donor-matched FMC63 CAR T cells showed similar levels of cytokine secretion across donors and nearly all E:T ratios (Figure 1D). CABA-201 elicited robust interferon gamma (IFNγ) production against CD19^+^ Nalm6 cells, which was associated with the potent cytotoxic activity (Figure 1D). Additionally, CABA-201 secreted high levels of effector cytokines associated with cytotoxic functions such as tumor necrosis factor alpha (TNFα), stimulatory factors known to potentiate activation and/or proliferation of the cells including IL-2, and the myeloid recruitment and maturation factor GM-CSF (Figure 1D). Cytokine levels were often higher in CABA-201 than in FMC63 CAR T cells; however, the differences, while statistically significant, were within one log_10_ decade (Figure 1D). The differences in cytokine production between the two CAR T cell constructs were significant, but we speculate that any biologic effects due to these differences will be minimal. Taken together, these data confirm that CABA-201 and FMC63 CAR T cells have similar or equivalent biological activity.

### Dose-related pharmacology

**Figure 2:**
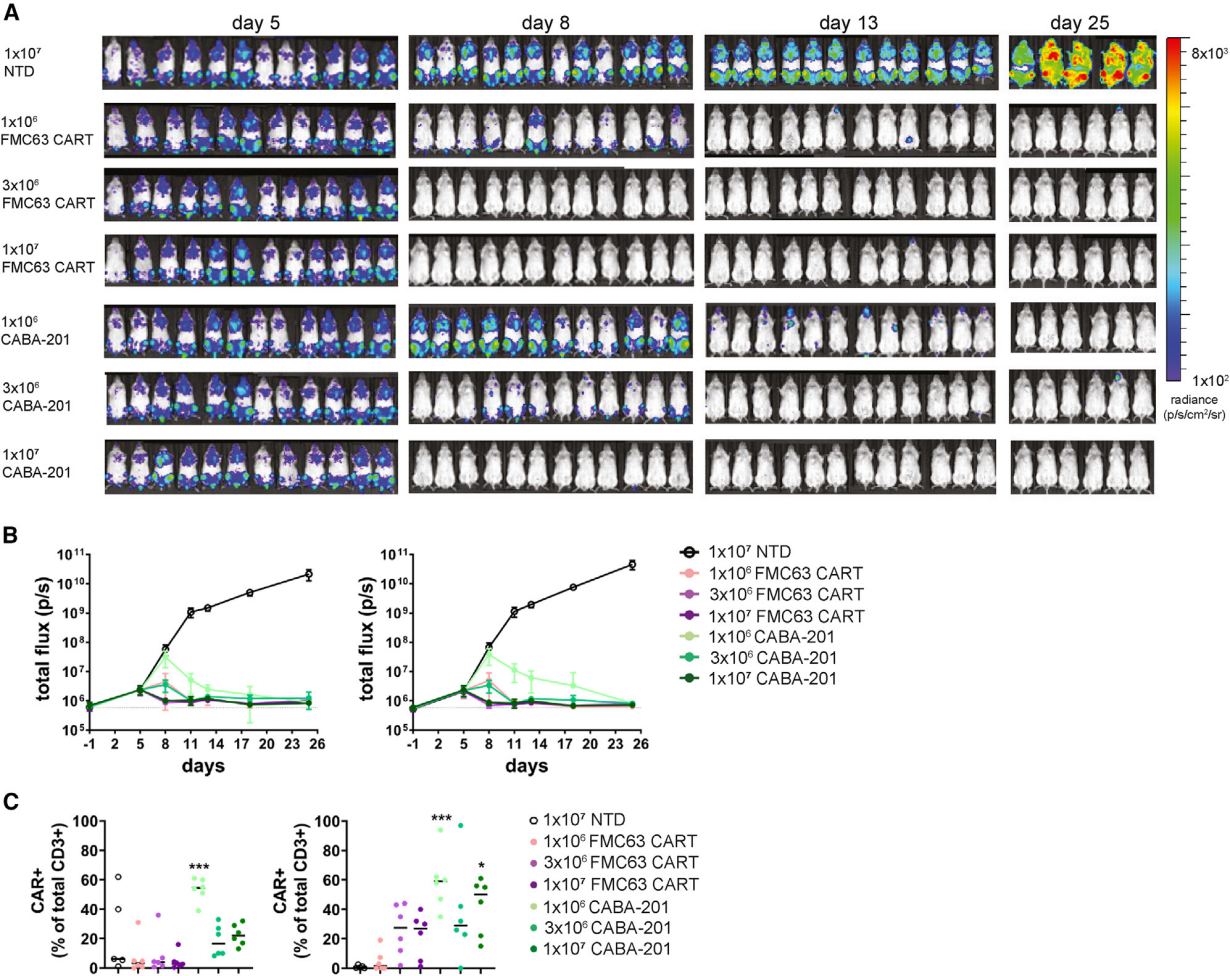
CABA-201 demonstrates activity related to dose *in vivo* Luciferase-expressing Nalm6 cells (1 × 10^6^ cells per mouse) were injected intravenously into NSG mice, followed 5 days later by injection with 1 × 10^6^, 3 × 10^6^, and 1 × 10^7^ CABA-201 or FMC63 CAR T cells and 1 × 10^7^ NTD T cells. Each experimental group consists of 12 mice. (A) Bioluminescence imaging quantification of Nalm6 CD19^+^ cells after FMC63 CAR T, CABA-201, or NTD treatment for days 5, 8, 13, and 25. (B) Ventral (left) and dorsal (right) bioluminescence (photons/sec) from days 1–25 after Nalm6 injection plotted as mean ± SD for each group. (C) Flow cytometric quantification of CAR^+^ T cells in peripheral blood (left) or spleen (right) at day 14 as mean ± SD for each group, calculated as a percent of the total human CD3^+^ T cell population measured are shown. Significance was determined by a one-way ANOVA analysis with Tukey’s post test to compare all groups. ∗∗∗∗*p* ≤ 0.0001, ∗∗∗*p* ≤ 0.001, ∗∗*p* ≤ 0.01, ∗*p* ≤ 0.05.

Although animal models of autoimmune diseases have been explored to elucidate disease mechanisms or evaluate disease-modifying agents, specifically small molecule or targeted biologic approaches, there are substantial limitations in validity, interpretation, and utility of such models for studying human CABA-201 cells. There are no well-established disease models for which a human cellular product(s) can be evaluated: *in vivo* testing of a human-based T cell product in rodents is inherently challenging. Human CAR T cell therapies will not engraft and persist long-term in immune-competent mice due to xenogeneic rejection. To date, humanized mouse models to circumvent these issues for exploration of CAR T cell products are hampered by variability in cellular source for engraftment (i.e., peripheral blood mononuclear cells [PBMCs] from autoimmune patients), residual murine myeloid cells, mediocre reorganization of human myeloid subsets, and reduced B cell re-constitution and antibody generation, all of which would directly impede a thorough and informative evaluation of the CD19-targeted CABA-201 cell product.^30^ For the *in vivo* testing of CABA-201, the core model utilized was an established tumor model (Nalm6-NSG) to demonstrate activity, toxicity, and/or biodistribution of CABA-201 T cell product. The Nalm6 B cell tumor line, which naturally expresses CD19, was transferred into mice and then followed 5 days later with infusion of CABA-201, FMC63 CAR T, or control NTD T cells. This model provides evidence of target engagement and enables testing of the human clinical products, as opposed to models requiring murine T cells, and it permits comparisons between CABA-201 activity to that of the clinically validated FMC63 CAR T cell (tisagenlecleucel) therapy. Activity of CABA-201 and FMC63 CAR T cells was assessed by bioluminescent imaging (BLI) of Nalm6 tumor cell growth kinetics (Figures 2A and 2B). FMC63 CAR T and CABA-201 CAR T cells significantly reduced Nalm6 tumor cell growth by day 8. A dose-dependent effect on tumor growth was observed in animals that received CABA-201 cells; a similar trend was apparent with FMC63 CAR T but was less prominent. CABA-201 cells at lower doses of 1 × 10^6^ and 3 × 10^6^ cells show slower kinetics of Nalm6 tumor control in early days when compared to FMC63 CAR T counterparts. However, by day 13, and for the remainder of the study period, tumor growth was controlled equally across all groups that received CD19 CAR T cells, irrespective of dose. At the termination of the study, only a minimal BLI signal was observed in two animals. The persistence of CD19 CAR T cells in tissues was evaluated, and CAR T cells were detectable in the peripheral blood and spleen of groups that received CABA-201 at day 14 (Figure 2C). At this time point, minimal numbers of FMC63 CAR T cells were present in the peripheral blood, but levels were higher in the spleen. Taken together, these results show that CABA-201 CAR T cells expanded, trafficked, and eliminated human CD19^+^ Nalm6 cells *in vivo*. Although there were slight variations in the kinetics of tumor elimination compared to FMC63 CAR T cells, CABA-201 performance in this model was equivalent (Figures 2 and S2). Overall, our data did not reveal any unexpected results and demonstrated the targeted, antigen-driven activity and tolerability of CABA-201.

### Off-target interactions of CABA-201

**Figure 3:**
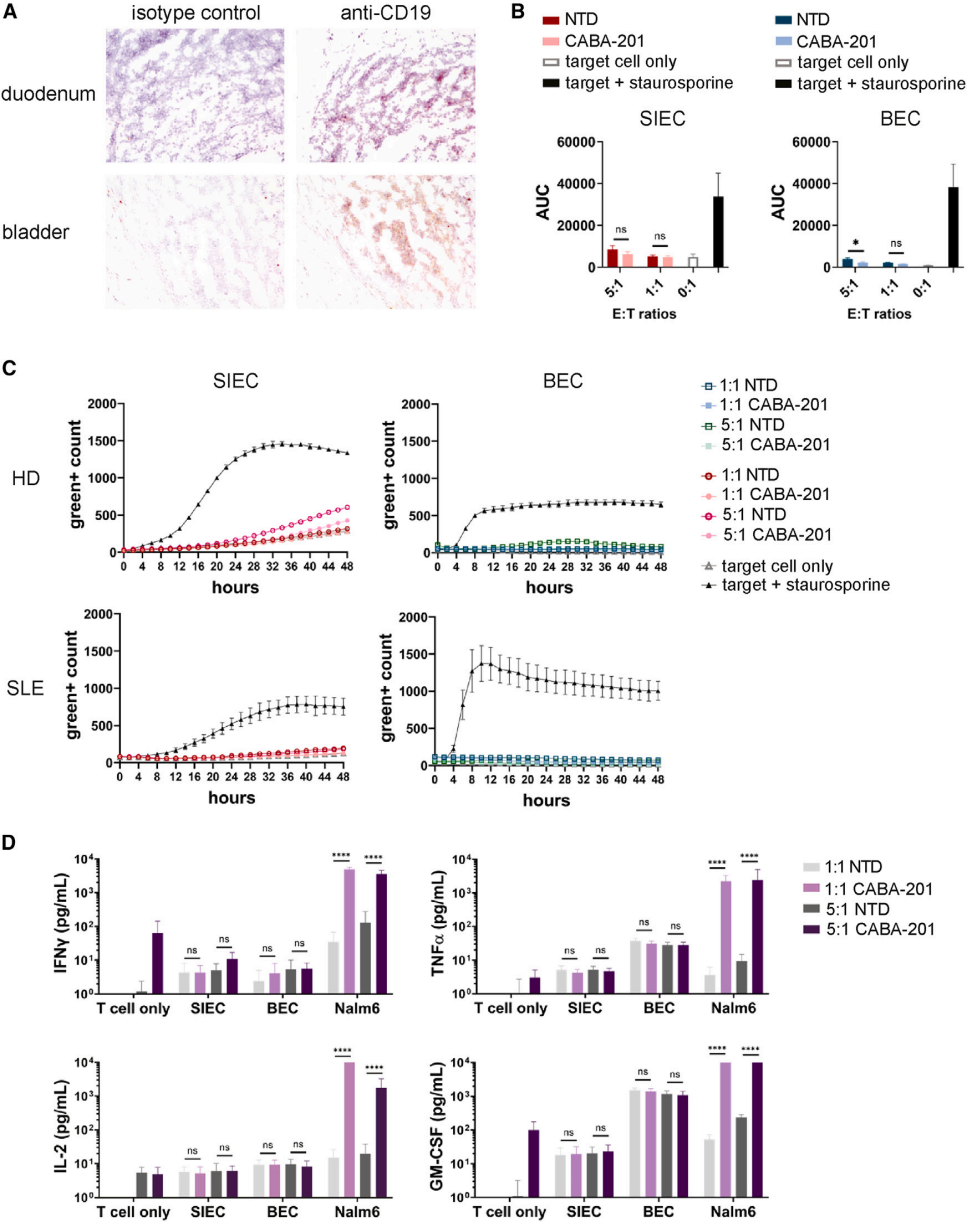
Assessment of CABA-201-induced cytotoxicity against bladder epithelial cells and small intestinal epithelial cells (A) Duodenum and bladder tissue sections from three independent donors were evaluated for IC78 anti-CD19 scFv binding and IgG1 Isotype binding. Representative histopathological immunohistochemistry images are shown. (B and C) Effector cells (CABA-201 and NTD T cells) from four different donors (three HD and one SLE) were co-incubated with normal healthy human primary small intestinal epithelial cells (SIECs) and bladder epithelial cells (BECs) for 48 h at indicated E:T ratios. Lysis was measured by incorporation of green dye over time via IncuCyte assay. Staurosporine was used as a positive control to induce the killing of SIECs and BECs. (B) AUC is shown as mean ± SD from four different donors. (C) Representative graphs are shown for HD and SLE donor. Similar results were obtained from the other donors. Green^+^ count is shown as mean ± SD in triplicates. (D) IFNγ, TNFα, IL-2, and GM-CSF production by CAR T cells in the supernatants collected after 48 h of CABA-201 from four donors co-cultured with normal healthy human primary BECs and SIECs. Donor-matched NTD cells are negative controls for the CABA-201 cells, and Nalm6 cells are positive controls expressing CD19. Each bar represents mean ± SD of four donors. Statistical differences between NTD and CABA-201 were determined by two-way ANOVA with Tukey’s multiple comparisons test. ∗∗∗∗*p* ≤ 0.0001, ∗∗∗*p* ≤ 0.001, ∗∗*p* ≤ 0.01, ∗*p* ≤ 0.05, ns = not significant. Experiments were repeated two times independently.

A membrane proteome array expressing approximately 5,000 proteins was used to assess binding specificity of the IC78 scFv, and no cross-reactive targets had been identified.^22^ To further evaluate the potential for interactions with anti-CD19 IC78 scFv, as well as to evaluate potential interactions against other membrane proteins that may not have been expressed within the membrane proteome array, a representative selection of 33 tissues was stained with a biotinylated anti-CD19 IC78 scFv. The human tissue cross-reactivity study showed that the biotinylated IC78 scFv bound to lymphocytes in expected human tissues, as well as the human bladder and duodenum (Figure 3A). Lymphocytes can perfuse multiple tissues in the body. They are present in abundance in lymphoid organs such as the lymph nodes, spleen, and thymus; however, they are also found both in intestinal tissue and in urothelial tissue. Because the data quality of the human tissue cross-reactivity study was limited by storage conditions of human tissues, false positive or negative results may have occurred, so additional studies were performed to evaluate the *in vitro* cytotoxicity and cytokine production of CABA-201 CAR T cells against primary human urothelial and small intestinal epithelial cells (SIECs). SIECs and bladder epithelial cells (BECs) were co-cultured with CABA-201 or their NTD counterparts produced from four matched T cell donors, including cells manufactured from SLE donor material (Figures 3B and 3C). Staurosporine was used as a toxicity control, and the functionality of the CABA-201 cells was confirmed via co-culture with CD19^+^ Nalm6 cells. Importantly, no CABA-201-induced cytotoxicity was observed in these two primary cell types. Furthermore, CABA-201 did not secrete IFNγ, TNFα, IL-2, nor GM-CSF at detectable levels following co-culture either with SIECs and BECs (Figure 3D) across the donors and E:T ratios tested. In summary, the results demonstrate the lack of specific reactivity of CABA-201 for healthy primary BECs and SIECs.

### Cytotoxicity toward intended target cells in autoimmune patients

**Figure 4:**
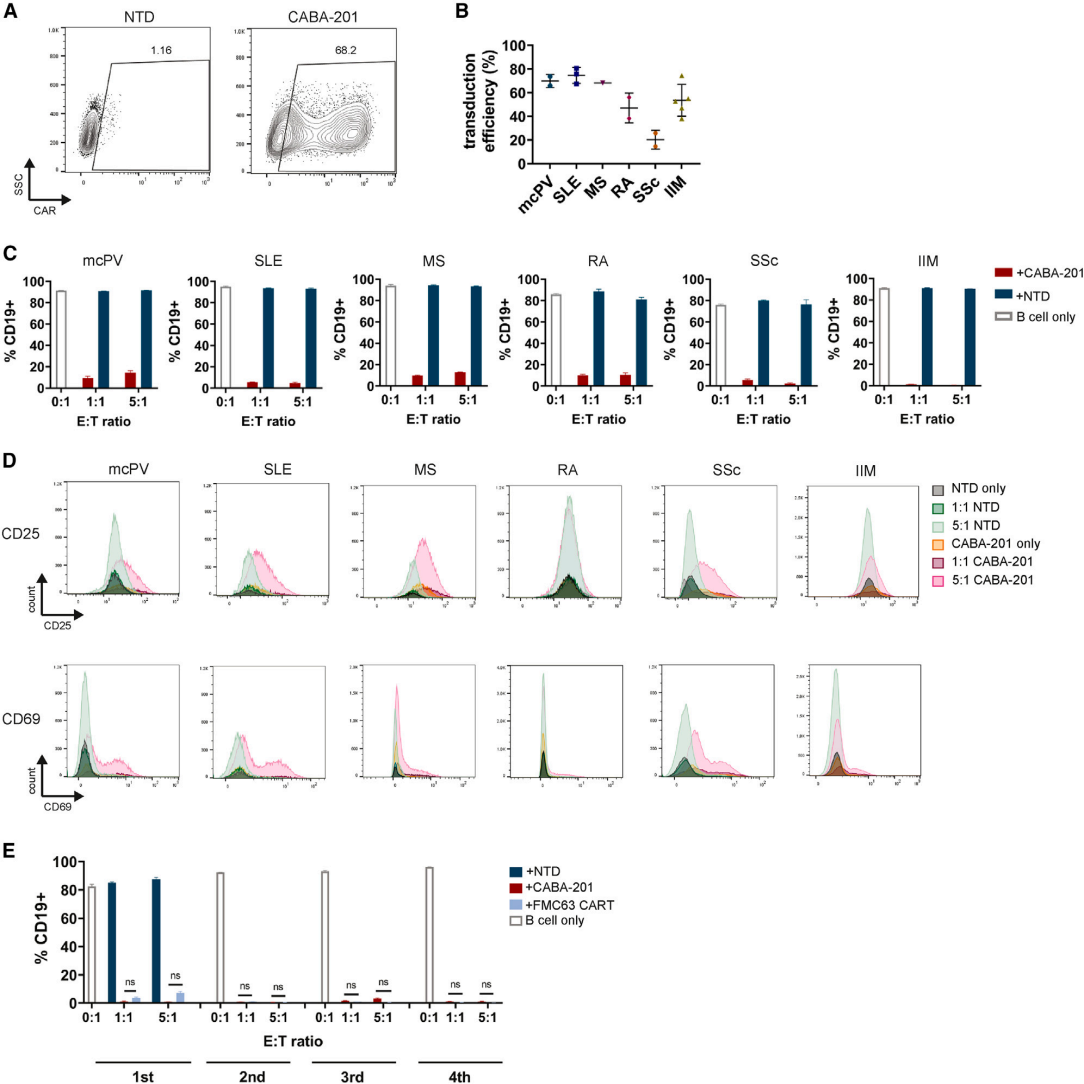
Cytotoxicity and activation of CABA-201 generated from patient T cells against autologous matched target B cells (A) Representative dot plots showing primary human T cells isolated from a multiple sclerosis (MS) patient expressing CABA19-IC78 CARs (CABA-201). (B) Summary graph showing transduction efficiency of primary T cells from multiple autoimmune disease patients. mcPV, mucocutaneous pemphigus vulgaris; SLE, systemic lupus erythematosus; RA, rheumatoid arthritis; SSc, systemic sclerosis; IIM, idiopathic inflammatory myositis. Mean ± SD is shown for each disease. (C) Effector T cells (CABA-201 or NTD T cells) generated from mcPV, SLE, MS, RA, SSc, and IIM donors were co-cultured with matched B cells isolated from the same patient at the indicated E:T ratios for 24 h. The percentage of CD19^+^cells is shown for each representative matched donor pair. Each bar represents mean ± SD of triplicates. (D) Representative histograms of CD25 (upper panel) and CD69 (lower panel) surface expression on effector T cells are shown for each representative matched donor pair following co-culture. 1:1 = E:T ratio of 1:1, and 5:1 = E:T ratio of 5:1. Experiments were repeated six times independently. (E) Effector T cells (CABA-201, FMC63 CAR T, or NTD T cells) manufactured from the SLE donor were co-cultured with matched B cells isolated from the same patient at the indicated E:T ratios for 24 h, and freshly isolated matched B cells were added every 24 h for three more rounds. NTD T cells were only included in the 1^st^ round of co-culture. The percentage of CD19^+^cells is shown for each round. Each bar represents mean ± SD of triplicates. Statistical differences between CABA-201 and FMC63 CAR T were determined by Mann-Whitney test. ns = not significant.

To demonstrate the activity and specificity of CABA-201 against intended target CD19^+^ B cells from autoimmune disease patients, we generated CABA-201 cells from a panel of autoimmune disease patient T cells. Primary human T cells isolated from autoimmune patient PBMCs demonstrate robust surface expression of CABA19-IC78 CAR across multiple patients and disease types (Figures 4A and 4B; Table S1). To investigate the activity and specificity of CABA-201 against intended target autoimmune B cells, we co-cultured autoimmune patient-derived CABA-201 CAR T cells with matched autologous B cells. Surface expressions of CD19 on B cells isolated from multiple autoimmune disease patients and disease types were similar to that of B cells isolated from healthy donors (Figure S3). Following 24 h of co-culture with patient-matched CABA-201 or NTD T cells, CABA-201 cells displayed a minimum of 90% of cytotoxic activity over the NTD and target-only controls across all indications, E:T ratios, and donors (Figure 4C). Furthermore, CABA-201 cells exhibited antigen-dependent activation as measured by CD69 and CD25 upregulation following co-culture with patient-matched CD19^+^ B cells (Figure 4D). Moreover, we assessed the serial killing functionality of CABA-201 cells manufactured from SLE donor material using a clinical manufacturing process by serially adding matched autologous B cells every 24 h (Figure 4E). CABA-201 cells displayed a minimum of 95% cytotoxicity over the NTD and target-only controls in all of 4 repeat additions of matched autologous B cells, and their serial killing functionality was comparable to donor-matched FMC63 CAR T cells. Together, the results demonstrate that CABA-201 cells manufactured from multiple autoimmune patient PBMCs show specific effector and cytotoxic functionality against the intended autologous target B cells.

## Discussion

The most compelling rationale for progressing anti-CD19 CAR T cell therapy in autoimmune disease came from recently published data in 15 patients with treatment refractory, moderate to severe, SLE, SSc, and idiopathic inflammatory myositis (IIM), following administration of an anti-CD19 CAR T cell comparable to the FDA-approved tisagenlecleucel. All patients exhibited dramatic, sustained clinical response and were able to remain off glucocorticoids and immunosuppressive medications. No moderate- or high-grade cytokine release syndrome or immune effector cell-associated neurotoxicity syndrome occurred. CAR T cells rapidly expanded and then contracted within 1 month post infusion. Patients experienced a transient suppression in their peripheral leukocyte counts, coincident with the chemotherapy preconditioning, while most B cell numbers remained suppressed for 2 to 5 months post infusion. All patients experienced a return of their B cells by 5 months, without disease recrudescence.^23^^,^^31^

We observed that CABA-201, a new anti-CD19 CAR T cell product candidate containing a clinically evaluated fully human CD19 binder (IC78) and 4-1BB costimulatory domain, is active against CD19^+^ target cells. Furthermore, CABA-201 has a comparable phenotype when manufactured using a clinical manufacturing process and similar level of cytolytic activity against CD19^+^ Nalm6 target cells compared to FMC63 CAR T (tisanglecleucel) across a variety of E:T ratios and different donors, including an SLE donor. Supernatants from co-cultures of CABA-201 and CD19^+^ Nalm6 cells were evaluated to further understand specific activity. CABA-201 manufactured from three independent healthy donors and one SLE donor produced expected increased levels of cytokines in response to their intended target cells, CD19^+^ Nalm6 cells, comparable to the levels of cytokines produced from similarly stimulated FMC63 CAR T cells.

For the *in vivo* testing of CABA-201, we utilized established tumor models (Nalm6) in NSG (NOD-*scid* IL2Rgamma^null^) mice to demonstrate activity and toxicity of the CABA-201 T cell product. Nalm6 tumor models have several caveats to consider. Firstly, they are not representative of human disease, as the CD19^+^ target cells do not differentiate into antibody secreting cells that produce disease-causing antibodies. Secondly, the target cells are tumor cells that grow rapidly and thus represent a more aggressive growth scenario that is not reflective of autoimmune CD19^+^ B cells *in vivo* and limits the duration of investigating activity. There exists the possibility that a subset of the CD19^+^ tumor cells may escape CAR T cell killing, which is a property that is not relevant to the human setting where B cells are not tumorigenic. Lastly, NSG or NCG mice may develop xenogeneic graft-versus-host disease (GvHD) due to human cell engraftment. This phenomenon can complicate the assessment of safety although the indicators of GvHD relative to CD19 CAR T cell-specific toxicity are discernable with appropriate analysis. Taking these limitations into account, the *in vivo* studies shown here establish the safety and activity of the anti-CD19 IC78 scFv, as expressed in CABA-201 cells (CABA19-IC78 CAR). Importantly, CABA-201 CAR T cell behavior was comparable to that of FMC63 CAR T (tisagenlecleucel) as demonstrated by these studies or as previously published.^22^ CABA-201, at lower testing doses, showed slower kinetics of CD19^+^ tumor cell line control than FMC63 CAR T cells; however, CABA-201 cells equally controlled the tumor growth by the end of the study period, and this slower kinetics was not observed in two independent experiments at a higher dose. Clinical experience suggests that allometric dose scaling from murine models has limited utility to predict clinically effective doses for human CAR T cell therapies, as cell quality influences long-term persistence once a threshold dose is achieved. Although none of the animal models reflect human disease, nor the dynamics of CABA-201 in the context of a complete human immune system, the models utilized do provide data relevant to safety and/or activity of the humanized anti-CD19 IC78 scFv binder and CABA-201 CAR T cells toward a CD19^+^ target cell line. In the *in vivo* efficacy study, the expansion and activity of CABA-201 T cells were comparable to that of the clinically validated FMC63-CAR T cells (possessing a murine anti-CD19 scFv and shared 4-1BB costimulatory domain) being used to currently treat SLE patients under a compassionate use protocol.^23^

A full cross-reactivity study was performed between the test article, a biotinylated anti-CD19 IC78 scFv, and normal, human frozen tissues of all major organs. Most tissue sections stained did not show anti-CD19 IC78 scFv binding. The negative tissues include adrenal gland, aorta, blood, bone marrow, breast, brain, cerebellum, cervix, colon, endometrium, esophagus, eye, fallopian tube, heart, kidney, liver, lungs, ovary, pancreas, pituitary, placenta, prostate, skin, spinal cord, skeletal muscle, testis, thymus, thyroid, and ureter. Five tissues had evidence of anti-CD19 IC78 scFv binding. These tissues include bladder, duodenum, lymph node, spleen, and thymus. The staining pattern described is consistent with lymphocyte staining, which is the intended target of the anti-CD19 scFv, as opposed to non-lymphocyte staining. Lymphocytes are present in abundance in lymphoid tissues such as the lymph nodes, spleen, and thymus. Furthermore, lymphocytes may be found both in intestinal tissue and in urothelial tissue as well. Out of an abundance of caution, primary urothelial (bladder) and small intestinal (duodenal) epithelial cells were selected to address the weak staining observed in the tissue cross-reactivity examination. CABA-201 cells did not interact with either primary urothelial (bladder) or small intestinal (duodenal) cells. There was no induction of dose-dependent CABA-201-specific cytolytic activity or cytokine production. Together, these studies further substantiate the specificity and affinity of the anti-CD19 scFv IC78 expressed by CABA-201 CAR T cells.

Finally, we sought to evaluate the cytolytic ability and activation of CABA-201 generated from the T cells of patients with autoimmune disease (mcPV, SLE, MS, RA, SSc, and IIM) toward the intended target CD19^+^ B cells isolated from the same patients. These patients constitute a cohort who have undergone various traditional treatments and are likely representative of the intended patient group for the CABA-201. Primary human T cells isolated from a panel of autoimmune patient PBMCs demonstrate robust surface expression of CABA19-IC78 CAR in multiple donors from different autoimmune disease indications. CABA-201 lysed intended autologous target B cells across all donors. Additionally, CABA-201 cells showed upregulated activation markers CD25 and CD69 when compared to their NTD counterparts as well, further supporting the effector functionality of autoimmune patient-derived CABA-201 cells. CABA-201 cells manufactured from SLE patient material using a clinical manufacturing process showed serial cytolysis of matched autologous B cells up to 4 times, highlighting their persistent cytotoxic capabilities. Taken together, these data demonstrated that multiple autoimmune patient T cells robustly express CABA19-IC78 CAR and show specific effector and cytotoxic functionality against the intended autologous target B cells, indicating that CABA-201 can be functionally manufactured from various autoimmune disease patient T cells.

In summary, the definitive preclinical studies presented here have supported an investigational new drug application enabling an open-label, phase 1/2 trial to evaluate the safety and preliminary efficacy of CABA-201 in patients with SLE, myositis, systemic sclerosis, and generalized myasthenia gravis. These data provide a foundation that may help inform the future development of CAR T cell therapies for patients with high unmet medical need in autoimmune disease.

## Materials and methods

### Cells

Nalm6 cells expressing enhanced green fluorescent protein (GFP) were produced by transducing the lentiviral encoding GFP to Nalm6 wild-type cells (ATCC). Nalm6 cells were sorted to select for high expression of GFP, and then the clonal population of cells was selected by serial limiting dilutions, and individual clones were selected for high expression of GFP. Human primary BECs were acquired from ATCC, and human primary SIECs were acquired from Cell Biologics. Leukapheresis from mcPV patients was obtained from an ongoing clinical trial (NCT04422912) after informed consent, primary PBMCs from SLE patients were obtained from Precision for Medicine, and MS RA, SSc, and IIM patient samples were obtained from Sanguine.

### Lentivirus production

For lentivirus production, VSV-G pseudotyped lentiviral particles were produced using a packaging system obtained from Aldevron. VPC cells were transfected at 4.7 × 10^6^ cells per mL in a sterile shake flask (Corning Costar) and transfected at a confluency of 90% with packaging plasmids pALD-VSV-G-K, pALD-Rev-K, and pALD-GAG/POL-K (Aldevron) plus pALD-CABA19-IC78-CAR (or other relevant lentivector) in a complex with LV-MAX Transfection Kit (Thermo Fisher). Lentivirus-containing supernatant was harvested after 48 h, filtered through a 0.45-μm polyethersulfone membrane (Genesee Scientific), and concentrated using ultracentrifugation at 62,976 × *g* for 108 min at 4°C and stored at −80°C. Virus titer was determined before use.

### CAR T cell production

CABA-201 cells and relevant controls (FMC63 CAR T and NTD) were manufactured using a previously reported protocol^32^ from healthy and SLE donor leukapheresis material (StemExpress). Briefly, T cells were selected/activated using anti-CD3/CD28 microbeads (Gibco) at a 3:1 ratio. Lentiviral vector that carries target CAR was added 24 h after activation, and T cells were expanded in the Xuri bioreactor (Cytiva) until day 9. The harvested cells were resuspended in the final formulation reagent CryoStor B/CryoStor 10 (BioLife Solutions) and then aliquoted as needed for preclinical studies. The drug product was then frozen to −90°C using a controlled rated freezer and stored at ≤−130°C.

For CABA-201 cells generated from patient samples, T cells (CD3^+^) were isolated from frozen patient PBMCs using the EasySep human T cell Isolation kit (StemCell Technologies) following manufacturer protocol. Isolated T cells were then stimulated with anti-CD3 and anti-CD28 beads (Dynabeads, Life Technologies) at a bead to cell ratio of 3:1. The culture medium was supplemented with 100 IU/mL interleukin-2 (IL-2). 24 h after stimulation, T cells were transduced with CAR lentivirus or mock transduced. Expansion of the T cells was monitored by measurement of cell size and concentration. Cells were counted every 40–56 h during the expansion by gently mixing cultures and collecting 100 μL of cells from culture volume and counted using NC-200 Automatic Cell Counter (Chemometec). T cells were harvested and frozen after 9 days in culture to use in subsequent functional assays.

### Isolation of primary human B cells

Primary human B cells were isolated from frozen patient PBMCs using the EasySep human B cell isolation kit (StemCell Technologies) following manufacturer protocol. Primary B cells and T cells were harvested from the same donor PBMCs to avoid allogeneic non-specific killing in the cytotoxicity assay.

### Measurement of cytotoxicity

#### IncuCyte-based assay

The ability of CABA-201 cells to kill CD19-expressing target cells was evaluated using IncuCyte assay. Briefly, Nalm6 cells endogenously expressing CD19 and modified to express GFP were co-cultured with CAR T or NTD T cells at E:T ratios of 5:1, 2.5:1, 1.25:1, and 0.625:1. E:T ratios were adjusted based on the expression level of CD19 CAR to match the transduced T cell to target cell ratio for CABA-201 and FMC63 CAR T cells. Co-cultures were monitored for 120 h using IncuCyte (Sartorius), and images were taken every hour with a 10× objective, and then GFP-positive cells were counted at each time point from 4 fields of images per well. The number of GFP-positive counts were the mean number of counts in each imaging field. GFP-expressing Nalm6 cells lose green count when Nalm6 cells are dying or dead.

For the *in vitro* off-target safety profiling of the CABA-201 CAR T cells, cytotoxicity co-culture experiments were performed with primary human healthy target cell types. Briefly, CABA-201, NTD T cells, and primary target cells (BECs and SIECs) were thawed and seeded in (coated) 96-well plates 24 h prior to co-culture. Immediately prior to initiating the co-culture, spent medium was replaced with fresh medium containing Caspase-3/7 green dye, and T cells were added to the target cells at different E:T ratios. To distinguish T cells from the primary target cells, T cells were labeled with an orange Cytolight dye (Sartorius). Co-cultures were monitored for 48 h using IncuCyte (Sartorius), and images were taken every 2 h with a 10× objective, and then green/GFP-positive cells were counted at each time point from 4 fields of images per well. The number of green/GFP-positive counts were the mean number of counts in each imaging field. Caspase-3/7 green dye-labeled cells are detected as green count when cells are dying or dead, whereas GFP-expressing Nalm6 cells lose green count when Nalm6 cells are dying or dead.

#### Flow cytometry-based assay

The ability of CABA-201 cells generated from isolated patient T cells to kill intended CD19^+^ target B cells from the same patient was evaluated using flow cytometry-based cytotoxicity assay. Briefly, primary human B cells isolated from autoimmune disease patient PBMCs were co-cultured with CABA-201 or NTD T cells from a matching donor at E:T ratios of 5:1 or 1:1. Prior to co-culture, T cells were thawed and allowed to recover in a 37°C humidified incubator with 5% CO_2_ for 16–20 h. After 24 h of co-culture, cells were labeled with CD3 and CD19 antibodies to distinguish T cells and B cells and then labeled with CD25 and CD69 to measure activation of the T cells. Loss of CD19^+^ B cells was used as a measurement of killing. Flow cytometry antibodies and reagents included the following: anti-human CD3 APC (clone: REA613), anti-human CD19 PE (clone: REA675), anti-human CD25 APC-Vio770 (clone: REA570), anti-human CD69 Vio Bright B515 (clone: REA824), and MACS Comp Bead kit, all from Miltenyi Biotec.

For flow-based serial cytotoxicity assay, CABA-201, FMC63 CAR T cells, and NTD T cells manufactured from the SLE donor using a clinical scale process were used. T cells were first thawed and allowed to recover in a 37°C humidified incubator with 5% CO_2_ for 16–20 h. To establish measurement of serial cytotoxicity, each condition was grouped into 4 sets, ranging from 1 to 4 rounds of target B cell co-culture. Primary human B cells were then isolated from matching SLE donor PBMC and mixed in with T cells as autologous targets as described in the previous section. After 24 h of co-culture, cells with 1 round of target stimulation were collected and labeled with antibodies for analysis. The remaining T cells were washed with fresh T cell media, and additional isolated B cells were mixed in as targets each 24 h, until 2, 3, and 4 rounds of target stimulation were achieved and collected for antibody labeling. Loss of CD19^+^ B cells after each round of re-introduction of B cells was used as a measurement of serial killing. Flow cytometry antibodies and reagents included the following: anti-human CD3 VioBlue (clone: REA613), anti-human CD19 APC (clone: REA675), and MACS Comp Bead kit, all from Miltenyi Biotec.

### Cytokine assays

CABA-201 T cells were co-cultured at E:T ratios of 5:1 and 1:1 with the CD19^+^ Nalm6 target cells or primary target cells (BECs and SIECs) for 24 h. Following co-culture, cell supernatants were harvested, and a multiplex cytokine assay was performed to quantify the secretion of 21 different soluble cytokines: *CCL3/MIP-1α*, *SDF-1α*, *IL-1b*, *IL-2*, *IL-4*, *IL-5*, *CXCL10/IP-10*, *IL-6*, *CXCL8/IL-8*, *IL-10*,*CCL1/Eotaxin*, *IL-12p70*, *IL-13*, *CCL5/RANTES*, *IFNγ*, *GM-CSF*, *TNFα*, *CCL4/MIP-1β*, *CCL2/MCP-1*, *CXCL1/GROα*, and *IL-18.* Negative controls consisted of donor-matched NTD cells co-cultured with the same targets and T cells cultured in the absence of target cell lines. Measurement of cytokines in the culture supernatants samples was performed with a Luminex bead array platform (Thermo Fisher) according to the manufacturer’s instructions. All samples were analyzed in triplicate and compared against multiple internal standards, with a seven-point standard curve.

### NSG animal model studies

All *in vivo* studies were performed under the spirit of GLP in an Association for Assessment and Accreditation of Laboratory Animal Care (AAALAC) accredited facility and under protocols approved by the Institutional Animal Care and Use Committee of Invivotek. During the study, the care and use of animals was conducted in accordance with the regulations of the AAALAC. NOD-scid IL2Rgamma null (NSG)/NOD.Cg-Prkdcscid Il2rgtm1Wjl/SzJ mice were from Jackson Laboratories. Nalm6 cells were transduced with lentiviral vectors encoding dual-reporter green fluorescent protein and luciferase (Nalm6 GFP-Luc) and sorted to enrich for CD19^+^GFP^+^ cells.

#### Experimental design

1 × 10^6^ Nalm6 GFP-Luc cells were injected intravenously into NSG mice (age 8 weeks), with 7 groups of 12 mice each after pre-treatment with 600 mg/kg intravenous immunoglobulin (IVIG, Creative Biomart) daily for 2 days. Infusion of IVIG to support Nalm6 engraftment was included for consistency with our previous studies.^31^ After 5 days, 1 × 10^6^, 3 × 10^6^, and 1 × 10^7^ FMC63-CAR T cells or CABA-201 cells or 1 × 10^7^ control NTD T cells were injected intravenously. Proliferation of Nalm6 cells was measured by BLI using the IVIS Spectrum. Bodyweights and animal health were assessed throughout the course of the study. Hematology was performed on days 13 and 27. The takedown on days 14 and 28 (day 25 for the NTD group) included flow cytometry analysis of blood and spleen.

#### Bioluminescence imaging

Bioluminescence was measured using the IVIS Spectrum imager (PerkinElmer). Up to five mice at a time were anesthetized with isoflurane and given a dose of 200 μL of a 15 mg/mL solution of D-luciferin by intraperitoneal injection (approximately 150 mg/kg). Imaging was performed 15 min following the injection. Bioluminescent images were acquired on day −1 (baseline) and days 5, 8, 11, 13, 18, and 25. Both dorsal and ventral images were acquired. Final BLI of all animals was performed on day 25 due to the health of the NTD group as a result of advanced tumor growth.

#### Flow cytometry of blood and spleen samples

Single-cell suspensions from freshly collected spleens were prepared by transferring individual tissues into gentle MACS C tubes containing 5 mL of RPMI. The C tubes containing the samples were placed onto the gentle MACS tissue dissociator (Miltenyi Biotec) to dissociate the tissue. The single-cell suspensions were filtered through 100-μm nylon filters (Falcon) and centrifuged. Following treatment with ACK buffer to lyse red blood cells, white blood cells and splenocytes were washed, counted, and resuspended in PBS buffer. Blood cells and splenocytes were stained with anti-huCD45 (clone HI30, BD Horizon), anti-huCD3 (clone SK7, Biolegend), huCD19 protein to detect the anti-CD19 scFv (Acro Biosystems), anti-huCD24 (clone ML5, Biolegend), and Live Dead (Invitrogen). All antibodies have previously been validated to be specific for human proteins and do not cross-react with mouse tissue. All data were analyzed using FlowJo software.

### Antigen density measurements

To estimate absolute number of membrane-bound CD19 on primary B cells, BD QuantiBRITE phycoerythrin (PE) bead kit was used following manufacturer instructions to generate a linear regression model during flow cytometry that can be used to estimate antibodies bound per cell (ABC) based on geometric mean fluorescence intensity (geoMFI). Primary B cells were isolated from healthy and various autoimmune disease donor PBMCs using EasySep Human Pan-B cell enrichment kit (StemCell Technologies) and then labeled with anti-human CD19 PE antibody (BioLegend, clone: 4G7) with a PE to antibody ratio of 1:1. The resulting geoMFI of the PE channel can be used to estimate ABC, or the number of surface CD19 antibodies per cell (CD19 density), using the linear regression model generated by QuantiBRITE PE beads. GeoMFI values were calculated using at least 10,000 PE^+^ events collected.

## Data and code availability

Data may be made available upon request.

## Supplemental information

Document S1. Figures S1‒S3 and Table S1

Document S2. Article plus supplemental information
