# Supplementary material for: Preclinical specificity & activity of a fully human 41BB-expressing anti-CD19 CART- therapy for treatment-resistant autoimmune disease
Source: Mol Ther Methods Clin Dev. 2024 May 20;32(2):101267. doi: 10.1016/j.omtm.2024.101267 (PMC11176803; doi:10.1016/j.omtm.2024.101267)
Supplement: Document S1. Figures S1‒S3 and Table S1 [file mmc1.pdf]

OMTM, Volume *32*

Supplemental information

Preclinical specificity & activity of a fully human 41BB-expressing anti-CD19 CART-therapy for treatment-resistant autoimmune disease

Binghao J. Peng, Andrea Alvarado, Hangameh Cassim, Soprina Guarneri, Steven Wong, Jonathan Willis, Julia SantaMaria, Ashley Martynchuk, Victoria Stratton, Darshil Patel, Chien-Chung Chen, Yan Li, Gwendolyn K. Binder, Rebecca Dryer-Minnerly, Jinmin Lee, and Samik Basu

A

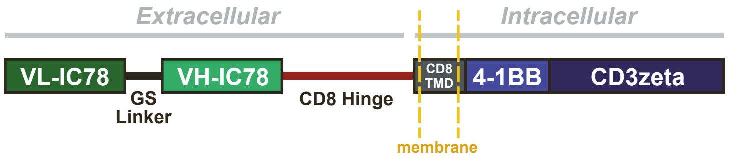

B

QAVLTQPPSVSEAPRQRVTISCSGSSSNIGNNAVSWYQQLPGKAPKLLIYYDDLLPSGVSDRFSGSKSGTSASLAISGLQSEDEADYYCAAWDDSLNGWVF
GGGTKVTVLGGGGGSGGGGSGGGGSEVQLVQSGAEVKKPGESLKISCKGSGYSFTSYWIGWVRQMPGKGLEWMGIIYPGDSDTRYSPSFQGQVTISADK
SISTAYLQWSSLKASDTAMYYCARLSYSWSSWYWDFWGQGTLVTVSS

Figure S1. CABA-201. (A) Schemetic of CABA-201 CAR construct. (B) Protein sequence of IC78 binder used in CABA-201 CAR construct.

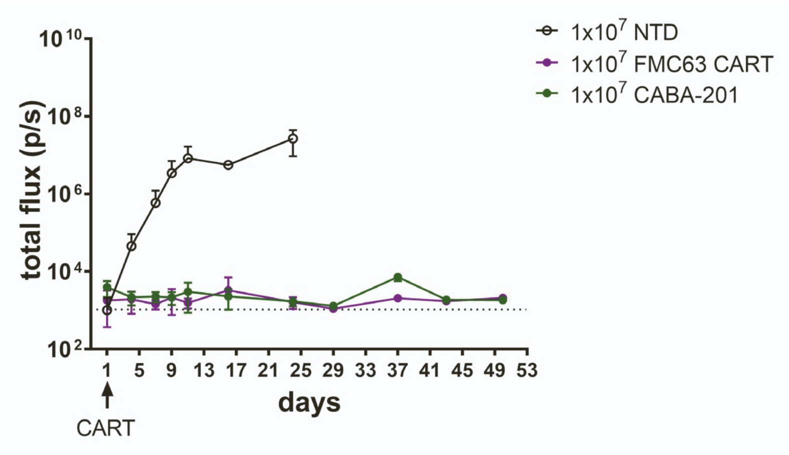

Figure S2. CABA-201 demonstrates activity in vivo. Luciferase-expressing Nalm6 cells (1×10^6 cells per mouse) were injected intravenously into NSG mice, followed 5 days later by injection with 1×10^7 NTD T-cells, FMC63 CART cells, or CABA-201 cells. Bioluminescence imaging quantification of Nalm6 $CD19^+$ cells after FMC63 CART, CABA-201 or NTD treatment for days 1, 4, 7, 9, 11, 16, 24, 29, 37, 43, and 50 were plotted as mean ± SD for each group (n=12 for NTD and n=18 for FMC63-CART and CABA-201 group). NTD T-cells treated group mice were terminated on day 28 for humane reasons.

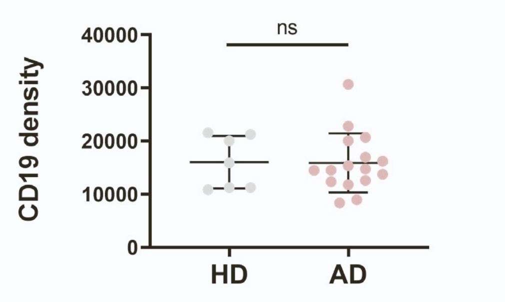

Figure S3. CD19 surface expression is comparable between healthy and autoimmune disease donors. CD19 surface expression on isolated B cells from healthy donors (n=7) and various autoimmune disease patients (n=16) were quantified using phycoerythrin (PE) quntification beads (see Methods). Each dot represents CD19 density (number of CD19 per cell) per donor. Mean ± SD are shown for HD (healthy donors) and AD (autoimmune disease patients). Autoimmune disease indication includes SLE (n=4), RA (n=9), and chronic inflammatory demyelinating polyneuropathy (CIDP, n=3). Statistical differences between HD and AD were determined by Mann-Whitney test. ns, not significant.

Table S1. Transduction and expansion of CABA-201 cells derived from autoimmune disease donors. Transduction efficiency of CAR and cell expansion during generation of CABA-201 cells from multiple autoimmune disease patient materials and types are shown. Donors with less than 5-fold cell expansions were largely due to poor quality of frozen PBMC samples.

Donor ID	Indications	Transduction Efficiency (%)	Cell expansion (fold)
mcPV Donor 1	Mucocutaneous pemphigus vulgaris	73.8	3.4
mcPV Donor 2		65.9	6.8
SLE Donor 1	Systemic lupus erythematosus	75.8	0.5
SLE Donor 2		67.5	5.22
SLE Donor 3		80.7	5.46
MS Donor 1	Multiple sclerosis	68.2	15.9
RA Donor 1	Rheumatoid arthritis	56.1	12.5
RA Donor 2		38.2	7.9
SSc Donor 1	Systemic sclerosis	14.6	7.4
SSc Donor 2		25.8	20.8
Myositis Donor 1	Idiopathic inflammatory myopathies	38.1	2.5
Myositis Donor 2		55.0	13.4
Myositis Donor 3		47.2	7.0
Myositis Donor 4		74.5	2.1
Myositis Donor 5		52.8	22.1
